# Volumetric and dosimetric impact of post-surgical MRI-guided radiotherapy for glioblastoma: A pilot study

**DOI:** 10.1259/bjro.20210067

**Published:** 2021-11-26

**Authors:** Marcus Tyyger, Suchandana Bhaumik, Michael Nix, Stuart Currie, Chandran Nallathambi, Richard Speight, Bashar Al-Qaisieh, Louise Murray

**Affiliations:** 1 Leeds Cancer Centre, Leeds Teaching Hospitals NHS Trust, Leeds, UK; 2 Department of Neuroradiology, Leeds Teaching Hospitals NHS Trust, Leeds, UK; 3 Radiotherapy Research Group, University of Leeds, Leeds, UK

## Abstract

**Objectives:**

Glioblastoma (GBM) radiotherapy (RT) target delineation requires MRI, ideally concurrent with CT simulation (pre-RT MRI). Due to limited MRI availability, <72 h post-surgery MRI is commonly used instead. Whilst previous investigations assessed volumetric differences between post-surgical and pre-RT delineations, dosimetric impact remains unknown. We quantify volumetric and dosimetric impact of using post-surgical MRI for GBM target delineation.

**Methods:**

Gross tumour volumes (GTVs) for five GBM patients receiving chemo-RT with post-surgical and pre-RT MRIs were delineated by three independent observers. Planning target volumes (PTVs) and RT plans were generated for each GTV. Volumetric and dosimetric differences were assessed through: absolute volumes, volume-distance histograms and dose-volume histogram statistics.

**Results:**

Post-surgical MRI delineations had significantly (*p* < 0.05) larger GTV and PTV volumes (median 16.7 and 64.4 cm^3,^ respectively). Post-surgical RT plans, applied to pre-RT delineations, had significantly decreased (*p* < 0.01) median PTV doses (ΔD99% = −8.1 Gy and ΔD95% = −2.0 Gy). Median organ-at-risk (OAR) dose increases (brainstem ΔD5% =+0.8, normal brain mean dose =+2.9 and normal brain ΔD10% = 5.3 Gy) were observed.

**Conclusion:**

Post-surgical MRI delineation significantly impacted RT planning, with larger normal-appearing tissue volumes irradiated and increased OAR doses, despite a reduced coverage of the pre-RT defined target.

**Advances in knowledge:**

We believe this is the first investigation assessing the dosimetric impact of using post-surgical MRI for GBM target delineation. It highlights the potential of significantly degraded RT plans, showing the clinical need for dedicated MRI for GBM RT.

## Background

Glioblastoma (GBM) is the most common malignant primary brain tumour in adults.^1^ Their aggressive nature and treatment resistance lead to poor prognosis, with a median survival of 12–15 months.^2^ The current standard of care is maximal safe tumour resection followed by radiotherapy (RT) with concurrent and adjuvant temozolomide.^3–5^ Current RT treatments rely on accurate gross tumour volume (GTV) delineations as tumour infiltration cannot be directly observed on anatomical MRI. As a result, isotropic margins of 20–30 mm are added to GTVs to create clinical target volumes (CTV).^3^ Therefore, to accommodate infiltration whilst minimising the volume of normal-appearing tissue irradiated, uncertainties in GTV delineation should be reduced wherever possible.

Current clinical guidance^3^ recommends a dedicated MRI for RT target delineation at the time of CT-simulation (pre-RT MRI). However, for RT departments with limited MRI access, there may be difficulty in acquiring an RT dedicated MRI. In these situations, it is common practice to include additional sequences for RT delineation on the <72 h post-surgical MRI acquired to assess the completeness of tumour resection. However, post-surgical acquisitions can contain acute oedema or inflammation, blood products and vascular changes around the surgical cavity. In addition, tumour progression or anatomical adjustment can occur during the delay between surgery and commencement of RT.^6–8^ Whilst clinical guidance^3^ acknowledges the risk when delineating based on the post-surgical MRI, it does not evaluate the potential severity of delineation inaccuracy or its dosimetric impact.

Previous studies assessed differences in high-grade glioma delineation between post-surgical and pre-RT MRIs, although results were inconclusive. Pennington et al^8^ found a statistically significant GTV increase of 11.09 cm^3^ when delineating on pre-RT MRI, concluding that tumour progression was the root cause. Conversely, Champ et al^9^ and Farace et al^10^ found no statistically significant changes in GTVs between MRIs.

To our knowledge, there remains no investigation assessing dosimetric differences between RT plans generated by post-surgical and pre-RT GBM delineations. This pilot study aims to quantify differences between delineations and their dosimetric impact in the context of GBM RT.

## Methods

A cohort of six patients (Table 1) with primary GBM treated with chemo-RT were enrolled within a separate local pilot study between May 2018 and September 2019 (IRAS Project ID: XXXXXX). One patient was excluded due to a lack of post-surgical MRI. Post-surgical and pre-RT MRIs were acquired within 72 h of surgery and prior to RT commencement, respectively. The median times from surgery to pre-RT MRI and planning CT were 42 days (range: 33 to 45) and 31 days (range: 28 to 34), respectively.

**Table 1.** Patient demographics, patient one was excluded as no post-surgical MRI was acquired

				Days after surgery to…		
ID	Sex	Age	Primary tumour location	Post-surgical MRI	CT-Simulation	Pre-RT MRI
Pt_2	M	48	(r)superior parietal lobe	1	28	34
Pt_3	M	55	(r)temporal lobe	3	32	45
Pt_4	F	66	(r)anteromedial frontal lobe	2	31	42
Pt_5	M	56	(r)parietal lobe	3	30	33
Pt_6	F	68	(l)posterior frontal lobe	3	34	45

Post-surgical MRIs were gadolinium contrast-enhanced T1W 2D spin echo sequences, acquired in the standard radiology position at 1.5 T (Aera, Siemens Healthineers, Erlangen, Germany) with: 2 mm contiguous slices, 1.2 × 1.0 mm in-plane resolution, 565 ms repetition time, 8.6 ms echo time and 250 Hz pixel^−1^ bandwidth.

Pre-RT MRIs were gadolinium contrast-enhanced T1W 2D spin echo sequences, acquired at 3 T (Prisma, Siemens Healthineers, Erlangen, Germany) in the RT treatment position with: 4 mm slices with 1.2 mm gaps between slices, 1.0 × 1.0 mm in-plane resolution, 600 ms repetition time, 6.0 ms echo time and 250 Hz pixel^−1^ bandwidth.

CT simulation (Brilliance Big Bore, Phillips Healthcare, Amsterdam, The Netherlands) was performed in the RT treatment position at 120 kVp with 1.17 × 1.17 mm in-plane resolution and 2 mm contiguous slices.

GTVs were independently delineated on post-surgery and pre-RT MRIs in RayStation (8B DTK, RaySearch Laboratories, Stockholm, Sweden), by a consultant neuroradiologist (CNR), a consultant oncologist (CCO) and a senior trainee oncologist (TCO). GTV was defined as the visible contrast-enhancing tumour and surgical cavity, following ESTRO-ACROP guidance.^3^ Observers were provided access to post-surgical radiology reports, and memory bias was accounted for by seven-day wait periods between delineations of individual patients. Clinical target volumes (CTVs) were generated from GTVs using 25 mm isotropic margins with manual adjustment for anatomical boundaries (*e.g.,* bone, falx cerebri, tentorium). Planning target volumes (PTVs) were grown from CTVs using 5-mm isotropic margins, with volumes clipped 5 mm from patient external contours for treatment planning purposes. Planning ‘PTV - 54 Gy OARs’ structures were created by subtracting brainstem, optic chiasm and optic nerves from PTVs. Organs at risk (OAR) were not specifically delineated for this study but instead the pre-existing clinical OAR delineations were used, previously contoured by the treating clinical oncologist and included: brainstem, cochleas, globes, lenses, lacrimal glands, optic chiasm, optic nerves and pituitary gland.

MRIs were rigidly registered to CT with registration quality assessed visually by a Clinical Scientist specialised in radiotherapy imaging. Target delineations were copied from MRI to CT. RT plans were generated in Monaco (v. 5.11.02, Elekta, Stockholm, Sweden) using the local glioma class solution. Treatment plans were optimised for individual target volumes from both MRI time points for each observer and patient. 60 Gy in 30 fractions 6 MV flattening filter free volumetric modulated arc therapy (VMAT) treatment plans were produced using a 180° coplanar arc and a 45° anterosuperior non-coplanar arc.

Dosimetric impact was assessed through differences in dose-volume histogram (DVH) statistics for the target and OAR constraints shown in Table 2 between post-surgical and pre-RT plans for each observer. PTV and ‘brain - PTV’ statistics for both post-surgical and pre-RT plans were determined using pre-RT MRI delineations (PTV_pre-RT_). These volumes were the closest available analogue to tumour and healthy brain tissue at the time of treatment, and were therefore used to represent ‘true’ tumour/normal tissue anatomy. Thus, the impact of the post-surgical plan on this ‘true’ anatomy could be assessed.

**Table 2.** Local dose volume histogram objectives for gliomas treated with 60 Gy in 30 fraction volumetric modulated arc therapy

Targets (Gy)		Mandatory OARs (Gy)		Optimal OARs (Gy)	
PTV	D99% > 54	Brainstem	D5% < 54	Lenses	D1% <6
	D95% > 57		Mean < 52	Lacrimals	D1% < 30
	59 < D50%<61	Optic Chiasm	D1% < 54	Cochleas	D50% < 45
	D5% < 63	Optic Nerves	D1% < 54	Brain - PTV	D10% < 57
	D2% < 64	Globes	D1% < 45		Mean < 24
				Pituitary	Max <45
PTV^*a*^	D99% > 51.3				
PTV - 54 Gy OARs^*a*^	D99% > 54				
	D95% > 57				

a Additional planning target volume (PTV) objectives were used where the PTV overlapped the 54 Gy organs at risk (OARs): brainstem, optic chiasm, and optic nerves.

Volumetric changes were determined through differences in absolute volume, contour similarity metrics and volume-distance histograms between post-surgical and pre-RT delineations for each observer. Dice Similarity Coefficient (DSC), sensitivity and specificity (equations 1-3) were calculated using pre-RT delineations as the reference contour and post-surgical delineations as the novel contour.^11^ DSC values ranged between 0 and 1, with values of 1 indicating the post-surgical and pre-RT delineations completely overlapped. Sensitivity values also ranged between 0 and 1, with a value of 1 meaning the pre-RT delineation was entirely contained within the post-surgical delineation. Specificity values ranged between -∞ and 1, with a value of 1 meaning the post-surgical delineation was entirely contained within the pre-RT delineation. Specificity values < 1 meant there were volumes of the post-surgical delineation outside the pre-RT delineation, and values < 0 meant these volumes were larger than the total pre-RT delineation volume.

Eq. 1 $$DSC=\frac{2.\left|ROI_{reference}\cap ROI_{novel}\right|}{\left|ROI_{reference}+ROI_{novel}\right|}$$

Eq. 2 $$Sensitivity=\;\frac{\left|ROI_{reference}\;\cap \;ROI_{novel}\right|}{\left|ROI_{reference}\right|}$$

Eq.3 $$Specificty=1-\;\frac{\left|ROI_{novel}\;not\;ROI_{reference}\right|}{\left|ROI_{reference}\right|}$$

**Figure 1.**
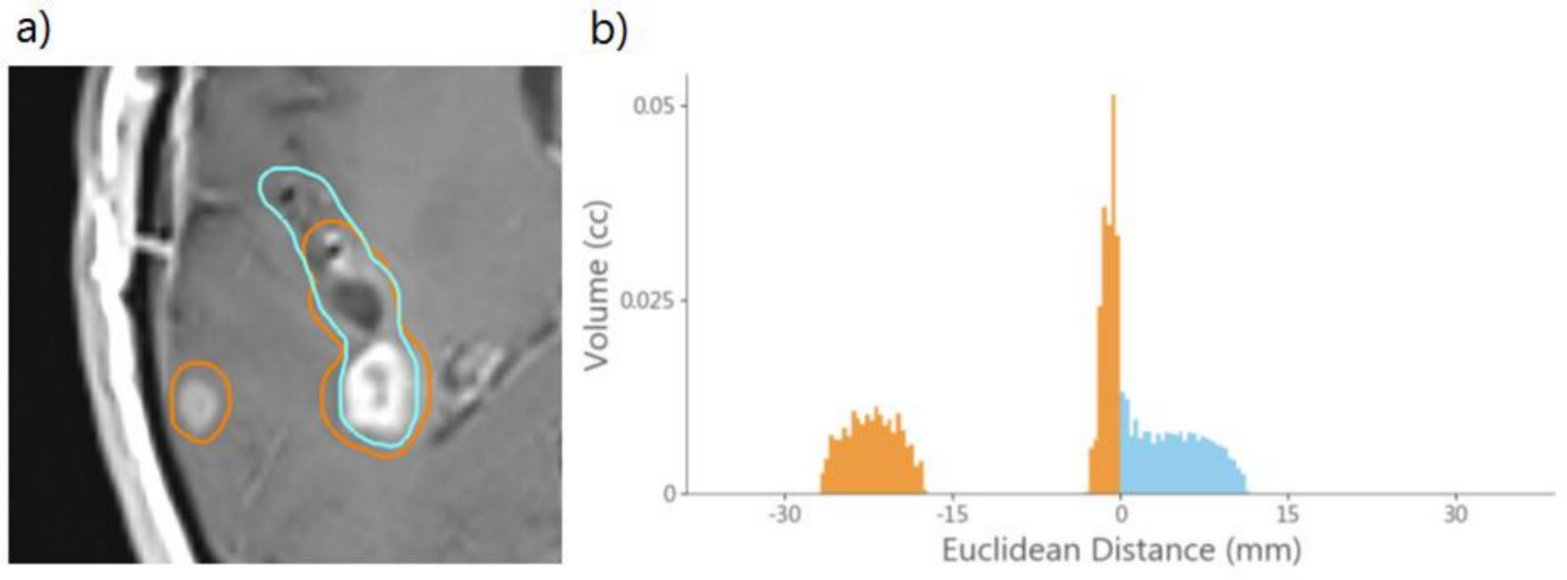
a) An example slice with pre-RT (orange) and post-surgical (blue) delineations. (**b**) The generated volume-distance histogram of the slice, with all union voxels omitted for display purposes. Distances > 0 are ‘extra’ voxels and are contained within the post-surgical delineation only, and distances < 0 are ‘missing’ voxels and are contained in the pre-RT delineation only. The extra volume is shown in blue and the missing volume in orange

Volume-distance histograms (Figure 1), based on a methodology by Nelms et al,^12^ were generated to allow further assessment of volumetric differences between delineations. These were discretised into individual voxels and classified as either ‘union’, ‘extra’ or ‘missing’. Union voxels were contained within post-surgical and pre-RT delineations. Extra and missing voxels were only contained within the post-surgical or pre-RT delineations, respectively. Missing and extra volumes were calculated by summing the overall number of missing and extra voxels, respectively. For all extra and missing voxels, the minimum Euclidean distance to the other delineation was determined, with union voxel distances set as zero. Missing voxel distances were set as negative, as this allowed them to be distinguished from extra voxel distances. Volume-distance histograms were then generated using the Euclidean distances and number of voxels for GTVs and PTVs, per patient, per observer. Cohort-level histograms for each observer were generated by summing all individual patient histograms, which allowed systematic changes to be identified.

Volumetric and dosimetric differences were statistically analysed in R^13^ using a linear mixed effects models through the ‘lme4’ package,^14^ with α = 0.05 as the threshold for statistical significance. Analysis of variance was used to assess differences between a null model only employing observers and patients as random effects, and a full model which also used MRI time point as a fixed effect.

## Results

**Figure 2.**
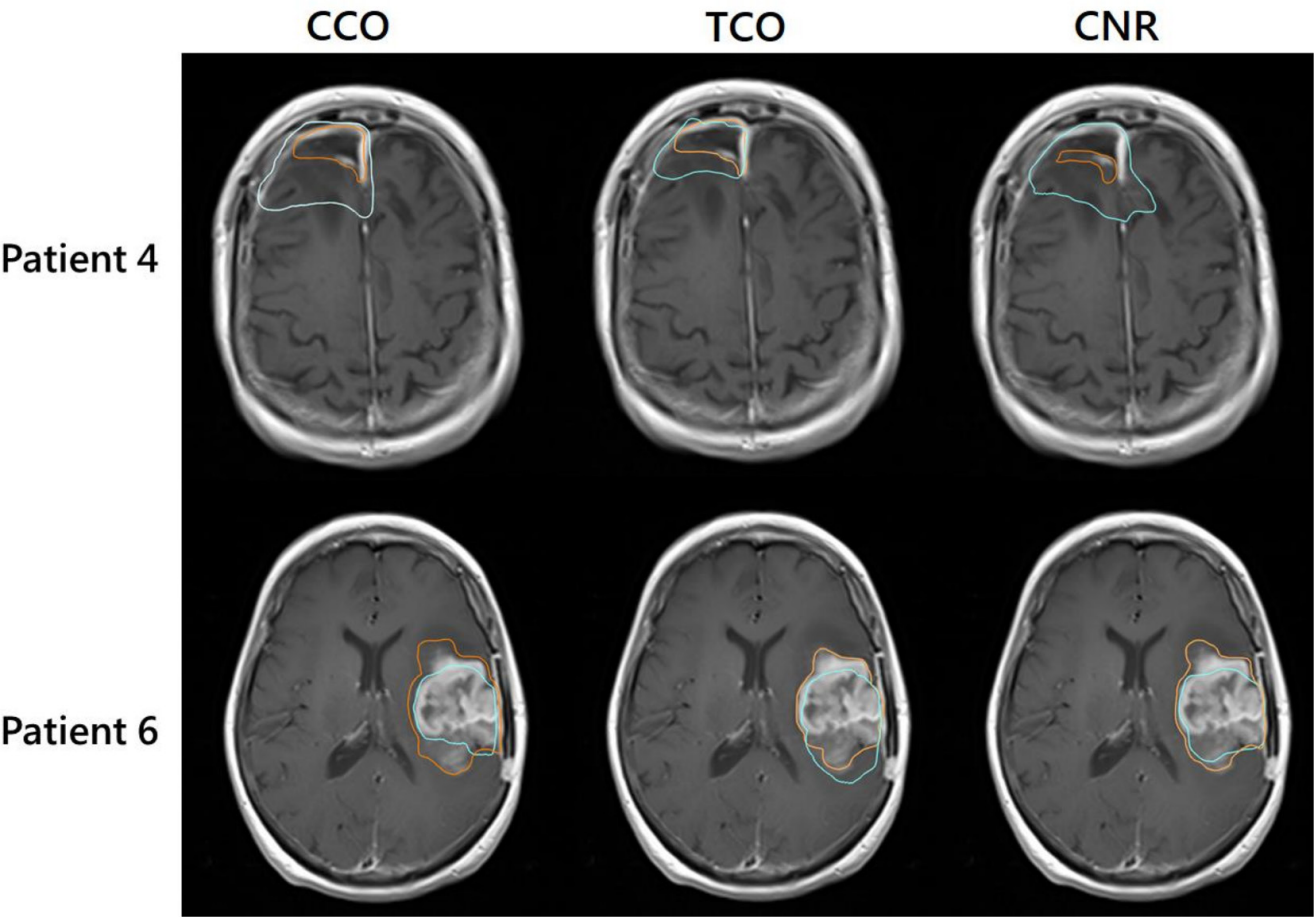
Axial slices of pre-RT MRIs for patients 4 and 6, with pre-RT (orange) and post-surgical (blue) delineations. Patient four showed the largest difference in GTVs, with post-surgical GTVs being larger than pre-RT for all observers. Patient six was the only patient that showed signs of progression between MRI acquisitions, and post-surgical GTV delineations were smaller than pre-RT GTVs for: consultant clinical oncologist (CCO), trainee clinical oncologist (TCO), and consultant Neuroradiologist (CNR)

**Figure 3.**
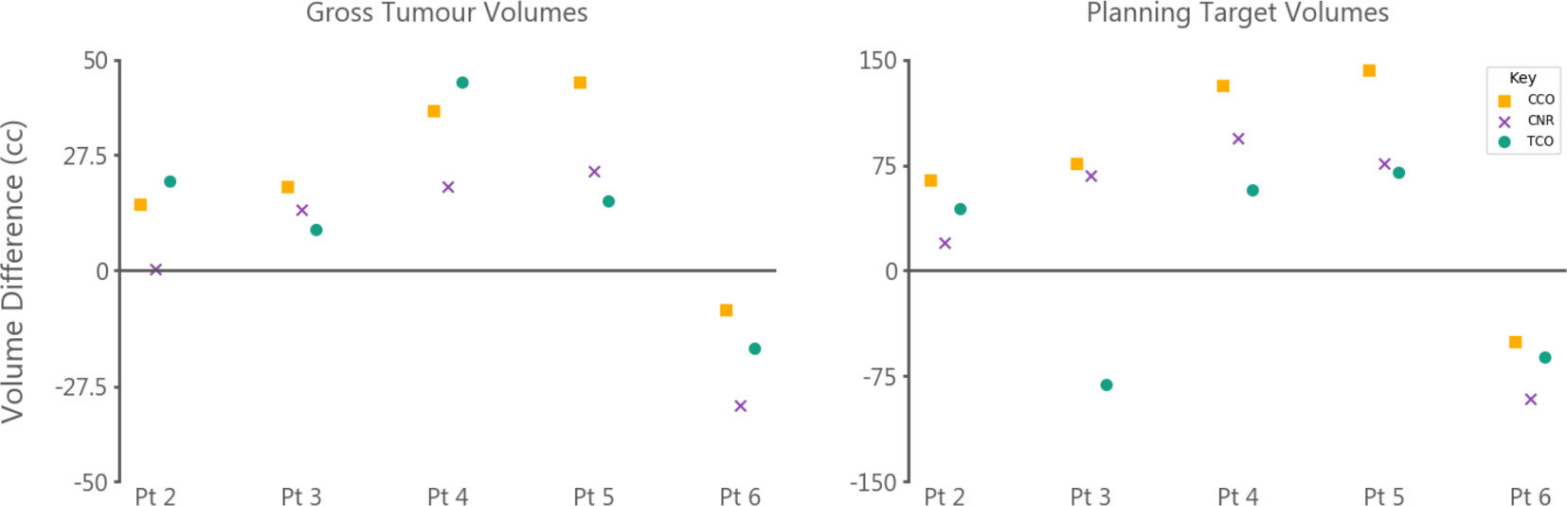
Absolute volume differences (cc) between post-surgical and pre-RT delineations for gross tumour and planning target volumes. Positive values indicate larger volumes on post-surgical MRI. Patient six showed progression between planning scans. TCO’s planning target volume for patient three was larger on pre-RT MRI despite a smaller gross tumour volume; this was caused by a small distant blood vessel being included in the gross tumour volume delineation and the large isotropic growth margins caused a large volume of normal appearing brain tissue to be included in the planning target volume. See Supplementary Material 1 for example images

Figure 2 shows post-surgical and pre-RT GTV delineations for patients 4 and 6. Patient four had the largest GTV reduction across observers on pre-RT MRI compared to post-surgical MRI. Patient six was the only patient to show signs of tumour progression between MRIs. Volumetric differences for GTVs and PTVs are shown in Figure 3. At a cohort level, across all observers, pre-RT delineations for GTVs and PTVs were smaller than post-surgical delineations by a median of 16.7 cm^3^ (range: −44.8 to 31.9, *p* value < 0.01) and 64.4 cm^3^ (range: −142.5 to 91.5, *p* value < 0.05), respectively. Patient six was the only patient to have larger delineations on pre-RT MRIs for all observers, with a median increase of 16.65 cm^3^ and 67.70 cm^3,^ respectively.

Contour similarity metrics were calculated with median values across all patients and observers shown in Table 3. Poor agreement between post-surgical and pre-RT GTV delineations was found in terms of DSC, sensitivity and specificity values. Whilst PTVs showed greater agreement than GTVs, overall the agreement was still poor.

**Table 3.** Median (range) values for contour similarity metrics for gross tumour volume (GTV) and planning target volume (PTV) delineations. Pre-RT and post-surgical delineations were used as the reference and novel contours, respectively

	DSC	Sensitivity	Specificity
GTV	0.58 (0.20, 0.76)	0.77 (0.53, 0.97)	0.02 (−3.82, 0.99)
PTV	0.80 (0.71, 0.92)	0.95 (0.80, 0.99)	0.80 (0.22, 0.98)

DSC, Dice Similarity Coefficient.

**Figure 4.**
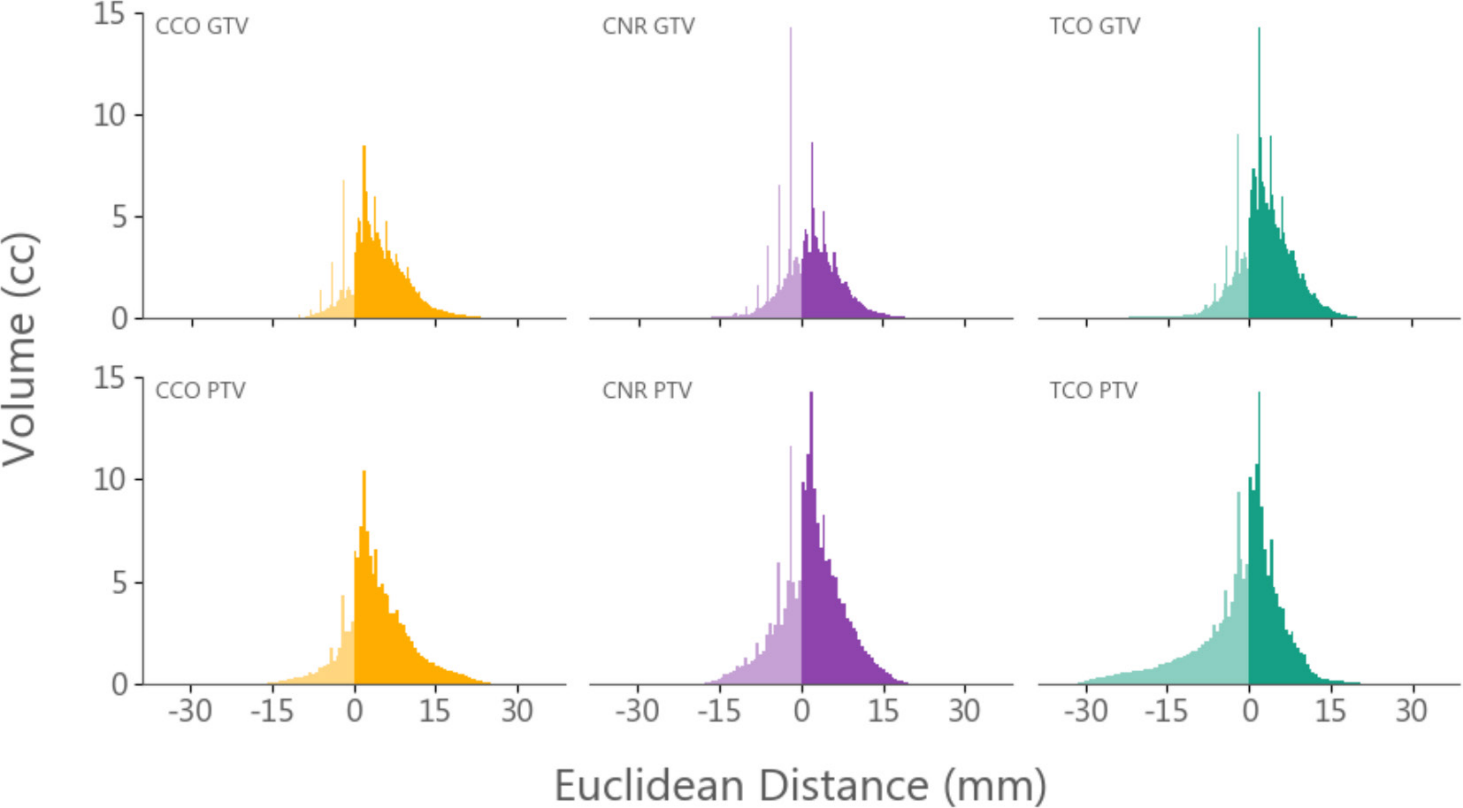
Cohort-level volume-distance histograms for GTVs and PTVs for each observer, generated by summing all individual patient histograms. Union voxels are omitted for display purposes. Distances > 0 are ‘extra’ voxels and are contained within the post-surgical delineation only, and distances < 0 are ‘missing’ voxels and are contained in the pre-RT delineation only. Extra volumes are shown in the dark colours, and missing volumes in the lighter colours

Volume-distance histograms were generated, with cohort-level histograms shown in Figure 4 and individual patient histograms in Supplementary Material 2. The median extra and missing volumes per patient for GTVs were 19.6 cm^3^ (range: 0.6 to 45) and 3.7 cm^3^ (range: 0.2 to 29.7), respectively. For PTVs these values were found to be 75.3 cm^3^ (range: 11.4 to 142.3) and 15.7 cm^3^ (range: 1.1 to 109.2), respectively.

**Figure 5.**
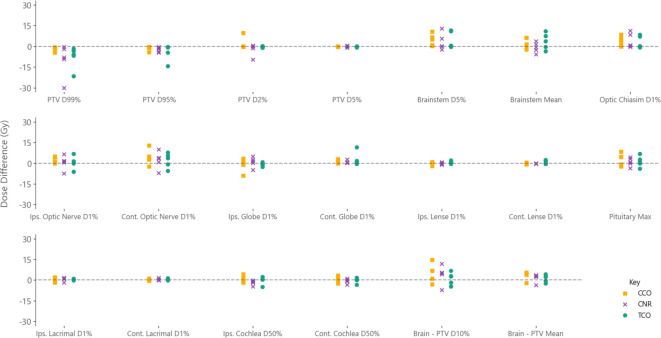
Dose differences for dose-volume histogram (DVH) statistics for all observers between post-surgical and pre-RT MRI based RT plans. Positive values indicate a higher dose on post-surgical MRI plan. Data points are individual patients for each observer, with observers shown in different colours and symbols. DVH statistics for planning target volumes (PTV) and ‘brain - PTV’ were determined using the pre-RT PTV volumes to give a representation of ‘true’ anatomy at the time of treatment for both post-surgical and pre-RT MRI. Ips: Ipsilateral. Cont: Contralateral. ON: Optic Nerve

RT plans were generated for all patients, including three patients who required compromised PTV coverage to achieve mandatory OAR constraints due to target volumes intersecting OARs. Figure 5 shows differences in DVH statistics between post-surgical and pre-RT plans for individual observers, based on the pre-RT delineations for PTV and ‘brain – PTV’ statistics, with positive values indicating a higher dose on post-surgical plans. At a cohort level OARs were found to have either a higher median dose on post-surgical MRI or a near zero difference with pre-RT MRI. These differences were not statistically significant except for ‘brain - PTV_pre-RT’_ D10% (5.3 Gy, range: −7.1 to 11.8, *p* value:<0.005), ‘brain - PTV_pre-RT’_ mean dose (2.9 Gy, range: −3.7 to 3.5, *p* value:<0.005), and brainstem D5% (0.8 Gy, range: −2.3 to 12.8, *p* value:<0.01). PTV_pre-RT_ D99% and D95% were found to be statistically significantly lower on post-surgical plans, with median values of −8.1 Gy (range: −30.0 to 0.4, *p* value:<0.01) and −2.0 (range: −4.5 to 0.3, *p* value:<0.01), respectively.

## Discussion

Dedicated planning MRI for RT target delineation is recommended in preference to the use of <72 h post-surgical MRI.^3^ MRI acquisitions shortly after surgery can contain oedema, ischaemia or inflammation around the surgical cavity, and the necessary delay between surgery and RT allows for post-surgical anatomical adjustment and tumour progression. However, for departments with limited MRI access, such as many of those within the UK,^15^ the resources required for a dedicated MRI scan may not be available. Previous studies into the impact of using post-surgical MRI for GBM delineation focused on volumetric differences and did not assess dosimetric impact. Here, we investigated the volumetric and dosimetric impact of RT target delineation on post-surgical MRI in a small cohort of primary GBM patients. Significant volumetric and dosimetric differences were found, showing degraded RT when delineating on post-surgical MRI.

Post-surgical MRI GTV and PTVs were larger than pre-RT MRI volumes across all observers, with median differences of 16.7 cm^3^ and 64.4 cm^3,^ respectively. Only one patient had smaller volumes on post-surgical MRI, attributed to tumour progression. For observer TCO, their patient three post-surgical PTV was smaller than their pre-RT PTV, despite the larger GTV on post-surgical MRI (Figure 3). This was caused by a small blood vessel far from the tumour bed being included in the pre-RT GTV, meaning the growth margins used caused a large volume of normal-appearing brain tissue to be included in the pre-RT PTV.

Volumetric results differ from previous investigations,^6,8,9^ which used larger patient cohorts. Pennington et al^8^ found post-surgical GTVs were smaller than pre-RT volumes due to tumour progression, whilst Champ et al^9^ and Farace et al^6^ found no statistically significant difference. The difference in results could be caused by the small cohort size paired with the heterogeneous nature of GBM or different timings between post-surgical and pre-RT MRI acquisitions. Recent investigations^16,17^ have identified potential GBM phenotypes with specific behaviours and characteristics. Therefore, a small cohort has potential to sample a smaller number of GBM phenotypes and not be representative of the full range of GBM behaviours and characteristics. It should be noted that Pirzkall et al^7^ found 53% of patients showed signs of progression between acquisitions, whereas in this investigation only one of five patients showed signs of progression.

Volume-distance histograms found overall GTV extra and missing volumes of 19.6 cm^3^ and 3.7 cm^3,^ respectively, across all observers. Thus, despite post-surgical delineations being significantly larger they did not entirely contain the pre-RT delineations. This result was also found through contour similarity metrics, where the median GTV sensitivity value was only 0.77. Whilst PTVs had a higher median sensitivity of 0.95 and a median DSC of 0.80, extra and missing volumes were still found (median 75.3 and 15.7 cm^3,^ respectively). Therefore, for this cohort of patients, post-surgical delineations did not accurately represent pre-RT delineations. Given the expected correlation between irradiated brain volume and toxicity,^18^ the increased treatment volume of RT plan guided by post-surgical MRI has the potential to cause additional toxicity. This may impact negatively on quality of life in a patient population who already have a guarded prognosis, although non-tumour complication probability models that examine this are not well established.

Dosimetric differences were assessed through DVH statistics, as seen in Figure 5, with insignificant differences found for most OARs. However, statistically significant median increases of 5.3 Gy, 2.9 Gy, and 0.8 Gy were found on post-surgical MRI for ‘brain - PTV_pre-RT_’ D10%, ‘brain - PTV_pre-RT_’ mean dose, and brainstem D5%, respectively. As the ‘brain - PTV_pre-RT_’ volumes were generated using pre-RT delineations for both post-surgical and pre-RT DVH statistics, the increase in doses can be attributed to larger PTV volumes on post-surgical MRI. Traditional late radiation-induced toxicity endpoints such as radiation optic neuropathy and brainstem necrosis are rarely observed in clinical practice when OAR constraints are met, perhaps as a result of the poor prognosis in this patient group. As such, differences in doses to these OARs are unlikely to be of clinical significance.

PTV DVH statistics for post-surgical and pre-RT plans were generated using the pre-RT PTV delineations to allow an assessment of the post-surgical plan on the ‘true’ anatomy at the time of treatment. Statistically significant decreases were found for PTV_pre-RT_ D95% and PTV_pre-RT_ D99% on post-surgical RT plans, with a median reduction at a cohort level of 2.0 Gy and 8.1 Gy, respectively. Observer CNR had a 30.0 Gy decrease in PTV D99% for patient two despite a small change in delineation volume. The large dosimetric change was due to the delineation difference being out-out-plane, meaning these regions were shielded by multi-leaf collimators and doses were actively minimised. Had this deviation been in-plane, it would not have been actively shielded and doses would also have been higher due to beam entrance/exit doses.

The origin of decreases in PTV_pre-RT_ D95% and D99% for post-surgical MRI RT plans is caused by regions of pre-RT target volumes not present in the post-surgical delineation; highlighting that despite larger treatment volumes on post-surgical MRI, RT treatments may not cover tumour extent at the time of RT.

Whilst we believe the cohort size is the main weakness of our investigation, other limitations should also be considered. Treatment planning was optimised for individual target volumes, meaning differing planning optimisation could have caused dosimetric differences. However, this effect was limited by using a single experienced clinical scientist to produce all plans, and optimised using the same priority order of mandatory OARs, target volumes and optional OARs.

As the pre-RT MRI sequence used thicker non-contiguous slices, there was potential for delineations to miss regions of tumour or to inaccurately represent the tumour tissue. However, as the delineation, volumes were large compared to the slice thickness the effect of interpolation should be small. We believe this effect could not be large enough to cause a median increase of approximately 16 cm^3^ in absolute volume.

As pre-RT MRIs were acquired on a 3 T MRI scanner, they had a higher signal-to-noise ratio (SNR) than the post-surgical MRIs. An increased SNR could have resulted in greater contrast at the boundary of the tumour, meaning delineations were less likely to account for uncertainty in the tumour boundary. Additionally, whilst visual assessment did not find any chemical-shift artefacts in the MRIs, the increased field strength without an increase in bandwidth means there was greater chemical-shift artefact on pre-RT MRIs.

It should also be noted that this investigation used a low number of observers from two separate disciplines, and the results from all observers were given equal weight. CNR is not trained clinically to define target volumes for RT, and TCO would have their delineations checked by a consultant clinical oncologist when used clinically. Therefore, these differences in delineation techniques and experience could have impacted the results.

## Conclusion

This pilot study assessed differences in target delineation and dosimetry between post-surgical MRI and pre-RT MRI in a small cohort of patients with GBM. Post-surgical GTVs and PTVs were found to be significantly larger than pre-RT delineations. Despite larger volumes, they did not necessarily contain the pre-RT delineations. Dosimetric analysis found insignificant changes for most OARs but significant increases for normal-appearing brain tissue and brainstem. Statistically significant dose decreases for PTV_pre-RT_ on post-surgical RT plans were also found, implying potential undercoverage of the ‘true’ tumour volume at the time of treatment. This work shows that tumour delineation based on post-surgical MRI can significantly impact RT planning for GBM, with larger volumes of normal appearing tissue being irradiated and target doses being significantly reduced. These dosimetric changes could affect outcomes, treatment tolerance and treatment-related toxicities. These results support clinical guidance that a dedicated pre-RT MRI should be used for target delineation in preference to the post-surgical MRI.
